# Solvent Bar Micro-Extraction of Heavy Metals from Natural Water Samples Using 3-Hydroxy-2-Naphthoate-Based Ionic Liquids

**DOI:** 10.3390/molecules23113011

**Published:** 2018-11-17

**Authors:** Philip Pirkwieser, José A. López-López, Wolfgang Kandioller, Bernhard K. Keppler, Carlos Moreno, Franz Jirsa

**Affiliations:** 1 Institute of Inorganic Chemistry, Faculty of Chemistry, University of Vienna, Waehringer Strasse 42, 1090 Vienna, Austria; Philip.pirkwieser@univie.ac.at (P.P.); wolfgang.kandioller@univie.ac.at (W.K.); bernhard.keppler@univie.ac.at (B.K.K.); 2 Department of Analytical Chemistry, Faculty of Marine and Environmental Sciences, Instituto de Investigación Marina (INMAR), University of Cádiz, 11510 Puerto Real, Spain; joseantonio.lopezlopez@uca.es (J.A.L.-L.); carlos.moreno@uca.es (C.M.); 3 Department of Zoology, University of Johannesburg, P.O. Box 524, Auckland Park, Johannesburg 2006, South Africa

**Keywords:** solvent bar micro-extraction, task-specific ionic liquids, heavy metal extraction, silver, cadmium, copper, lead, drinking water, sea water

## Abstract

Developments in the liquid micro-extraction of trace metals from aqueous phases have proven to be limited when extended from pure water to more complex and demanding matrices such as sea water or wastewater treatment effluents. To establish a system that works under such matrices, we successfully tested three task-specific ionic liquids, namely trihexyltetradecyl- phosphonium-, methyltrioctylphosphonium- and methyltrioctylammonium 3-hydroxy-2-naphthoate in two-phase solvent bar micro-extraction (SBME) experiments. We describe the influence of pH, organic additives, time, stirring rate and volume of ionic liquid for multi-elemental micro-extraction of Cu, Ag, Cd and Pb from various synthetic and natural aqueous feed solutions. Highest extraction for all metals was achieved at pH 8.0. Minimal leaching of the ionic liquids into the aqueous phase was demonstrated, with values < 30 mg L^−1^ DOC in all cases. Sample salinities of up to 60 g L^−1^ NaCl had a positive effect on the extraction of Cd, possibly due to an efficient extraction mechanism of the present chlorido complexes. In metal-spiked natural feed solutions, the selected SBME setups showed unchanged stability under all conditions tested. We could efficiently (≥85%) extract Cu and Ag from drinking water and achieved high efficacies for Ag and Cd from natural sea water and hypersaline water, respectively. The method presented here proves to be a useful tool for an efficient SBME of heavy metals from natural waters without the need to pretreat or modify the sample.

## 1. Introduction

In view of the ongoing pollution of waterbodies with heavy metals from anthropogenic sources and the rise of emerging pollutants such as silver [1,2,3], research in adapting extraction methods to meet these challenges is increasingly important [4]. One demanding task is to extract trace metals from natural waters: For these oftentimes complex sample matrices, traditional liquid- or solid-phase extraction methods may require difficult sample treatment, acidification, and additional additives for complexation or large volumes of environmentally harmful solvents [5]. In recent years, growing attention has therefore been given to replacing toxic organic solvents and miniaturizing processes to attain greener extraction techniques [6]. Several studies have incorporated micro-extraction approaches, highlighting liquid- or solid-phase micro-extraction setups for trace metals [7,8]. Despite their potential for green extraction, however, liquid micro-extraction methods still require improvement for their application to natural- and particularly to saline waters. This is due to the lack of efficient extractants and organic solvents that offer sufficient stability during extraction [9,10].

Recently, ionic liquids (ILs), which are salts in the liquid state below 100 °C, have been included in liquid micro-extraction of metals because of their high thermal stability and low vapor pressure [4,11]. Especially the subgroup of task-specific ionic liquids (TSILs) has demonstrated great potential in this regard: they may contain metal ion chelating groups and therefore simultaneously function both as hydrophobic solvents and extractants [12,13]. Adding functional groups to anions of ILs proved to be successful in various cases, e.g., by implementing thiosalicylate and thioglycolate groups to extract Cd, Co and Zn [14,15], maltolate-based TSILs to remove several radionuclides [16], or phosphinate TSILs for the separation of yttrium [17]. Nevertheless, liquid-liquid extraction methods generally are hampered by the partial solubility even of highly hydrophobic ILs [18,19]. This so-called leaching still prevents an economical and environmentally friendly application of TSILs as a greener alternative to existing extracting agents [4].

In order to minimize leaching, solid supports for ILs have been proposed as a feasible solution [4]. Simultaneously, the unique physico-chemical properties of ILs drew interest for application in micro-extraction methods [20,21]. Among the benefits of using TSILs in liquid micro-extraction are their above-mentioned non-volatile nature and high viscosity. Both attributes help overcome instability problems of conventional organic solvents [22]. Hollow fiber liquid phase micro-extraction (HFLPME) is one immobilization strategy that has been used in trace metal extraction and analysis [23,24]. HFLPME can be set up in the form of a solvent bar (solvent bar micro-extraction, SBME), in which the organic solution of ionic liquid is supported in the fiber pores and lumen [25,26]. In this approach, the fiber may be left floating freely in the sample during extraction, considerably simplifying the procedure. Successful applications of ILs in HFLPME, including SBME, have been used to preconcentrate Ni from sea water [10] and biological samples [27]. Additional successes include the use of IL methyltrioctylammonium chloride to preconcentrate Ag from sea water [28] or, most recently, the use of several TSILs in multi-elemental extractions, including Cr and Pt [29].

In SBME the immobilized organic phase should offer high extraction efficacies while being non-volatile and insoluble in water [25]. These favorable properties have been demonstrated for the recently synthesized TSILs trihexyltetradecylphosphonium 3-hydroxy-2-naphthoate ([P_66614_][HNA]), methyltrioctylphosphonium 3-hydroxy-2-naphthoate ([P_1888_][HNA]) and methyltrioctylammonium 3-hydroxy-2-naphthoate ([N_1888_][HNA]). Moreover, these TSILs show a high affinity towards several heavy metals, including Cu, Ag, Cd and Pb, in liquid-liquid and solid-liquid extractions [30]. We implemented these three TSILs in SBME experiments in order to improve their stability and consequently their suitability for application in micro-extraction methods.

Hence, the aim of this work was to apply the three TSILs in a simultaneous multi-elemental two-phase SBME of the metals Cu, Ag, Cd and Pb from natural waters, including complex matrices such as sea water and a hypersaline brine. The developed setup should enable a simple, highly efficient and environmentally friendly method for metal separation. To meet this aim, the study was divided into three consecutive parts:

(1) Based on earlier results [30], we evaluated the influence of organic additives, pH, extraction time, stirring rate and volume of ionic liquid on extraction efficacy and leaching. The parameters that achieved the most promising results were selected for subsequent experiments to:
(2) Study the influence of different salinities on the simultaneous extraction of the four metals from synthetic samples.
(3) Study the applicability for the extraction of the four metals from metal-spiked natural feed solutions, including drinking water, sea water, hypersaline water and a wastewater treatment plant effluent.

Using recently synthesized TSILs for the first time in a SBME setup is a step forward in developing simple and ecofriendly methods to directly extract heavy metals from natural waters without sample modification or pretreatment.

## 2. Results and Discussion

### 2.1. Influence of Physico-Chemical Properties on SBME

In the following, we describe the effects of varying physico-chemical parameters in the experimental setup and summarize the impact of each parameter on the performance of SBME of the metals Cu, Ag, Cd and Pb.

#### 2.1.1. Organic Additives

**Figure 1:**
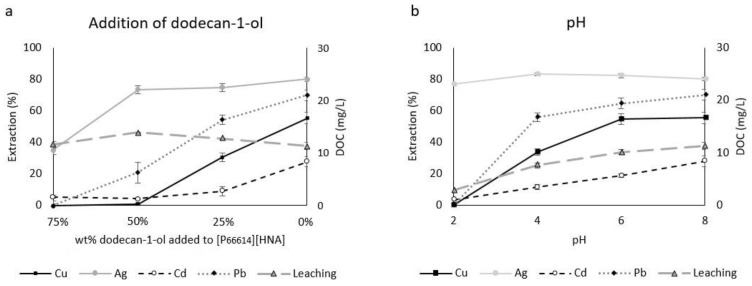
Effects of (**a**) adding dodecan-1-ol at pH 8.0 and (**b**) the pH using pure [P_66614_][HNA] on extraction efficacy and leaching for an extraction time of 2 h (n = 3, error bars = ± SD).

Organic additives are generally used in SBME to increase the diffusion of metals into the fiber lumen when utilizing viscous TSILs. They are therefore an important factor in improving SBME setups [28]. All results regarding the influence of organic additives on the three TSILs used are shown in Table S1; for [P_66614_][HNA] and dodecan-1-ol the results are additionally depicted in Figure 1a. In the case of [P_66614_][HNA], the viscosity of the pure TSIL was previously determined as 719 cP [30], which is reduced by adding less viscous dodecan-1-ol (16.1 cP [31]). Nevertheless, no clear negative correlation between extraction efficacy and concentration of organic additive was evident. In particular, Cu, Cd and Pb extraction increased significantly between 25 wt % dodecan-1-ol and the pure TSIL [P_66614_][HNA], with the latter offering the highest efficacies.

Ag extraction for [P_66614_][HNA], however, behaved differently: it was the only metal showing moderate extraction (35.2 ± 2.9%) at 75 wt % dodecan-1-ol and higher extraction (75–80%) at all lower concentrations of dodecan-1-ol. No significant differences were achieved for 50%, 25% and 0% dodecan-1-ol. The simultaneous extraction of the four metals using pure [P_66614_][HNA] yielded the following ranking of efficacies: Ag (80.2 ± 1.2%) > Pb (70.1 ± 3.4%) > Cu (55.5 ± 3.7%) > Cd (27.9 ± 3.2%). Egorov et al. described varying efficacies for different metals for methyltrioctylammonium salicylate, a TSIL similar to the compounds used in this study. Metal ions were extracted as salicylate complexes based on the stability constants of the respective metal-salicylate complexes [32]. Additionally, Platzer et al. attributed differences in extraction efficacies for metal ions that depend on the same functional anion to the stability of the respective metal aquo complexes M(H_2_O)_4_^2+^ [33]. Both described effects could be relevant to the results presented here. Although the prevalent extraction mechanisms have not been clarified experimentally, ILs are known to employ an ion exchange and a neutral extraction (ion pairing) mechanism, both in competition, depending on sample composition and properties of the respective IL as described by Janssen et al. [34] and Dietz et al. [35]. TSILs incorporating hydrophobic cations with long alkyl chains, like the ones used in this work, were reported to dominantly extract metals based on ion pairing rather than cation exchange [35,36].

For [P_1888_][HNA] and [N_1888_][HNA] the lowest concentration of 50 wt % dodecan-1-ol performed best. The setup using [P_1888_][HNA] achieved extraction efficacies of 87.4 ± 0.8% for Cu and 50.4 ± 2.1% for Pb. Using [N_1888_][HNA], 90.1 ± 1.7% of Cu and 69.1 ± 2.7% of Pb could be extracted, whereas Ag and Cd were not extracted efficiently with either TSIL.

Our observations on the effects of adding organic solvents to the TSILs suggest that extraction behavior could not be improved by reducing the viscosity, which is in contrast to the results for methyltrioctylammonium chloride reported by López-López et al. [28]. Leaching values (Figure 1a) also favor the use of pure TSIL [P_66614_][HNA], which showed the lowest dissolved organic carbon (DOC) value of 11.3 ± 0.7 mg L^−1^. Overall, our data showed very low leaching when compared to previous studies using TSILs in similar setups with polypropylene fibers [15,29]. Additionally, our experiments achieved a reduction by approx. 40% when compared to liquid-liquid extractions using identical TSILs under similar conditions [30]. Leaching for [P_1888_][HNA] and [N_1888_][HNA], which is summarized in Table S2, increased with decreasing dodecan-1-ol concentration. Using a 50 wt % dodecan-1-ol solution, the leaching for [P_1888_][HNA] was 19.4 ± 0.6 mg L^−1^ DOC and for [N_1888_][HNA] 20.6 ± 0.2 mg L^−1^ DOC. Based on the very low solubility of dodecan-1-ol in water at 25 °C (4 mg L^−1^ [37]), we assume that most of the DOC is attributable to dissolved TSIL. The before mentioned prevalence of a neutral extraction mechanism as opposed to cation exchange for hydrophobic TSILs could explain the overall low leaching values obtained in this work. However, this has to be confirmed in further studies by determining the contribution of cation and anion to the DOC and combining this data with e.g., UV measurements as previously conducted by Messadi et al. [38].

The highest extraction efficacies combined with high stability were achieved using pure [P_66614_][HNA] and the two respective 50 wt % dodecan-1-ol-TSIL mixtures. We therefore selected these for the subsequent experiments. To enhance readability of the text, the mixtures of [P_1888_][HNA] and [N_1888_][HNA] with 50% dodecan-1-ol are abbreviated in the following as [P_1888_][HNA]-DdOH and [N_1888_][HNA]-DdOH, respectively.

#### 2.1.2. Feed Solution pH

pH can influence the extraction mechanisms of ILs, namely the balance of ion exchange and neutral extraction [34]. On the other hand, changes in metal speciation at varying pH values, e.g., distinct equilibria of differently charged metal aquo- or hydroxide species [39], may alter the uptake by the IL. The results for SBs with [P_66614_][HNA] (Figure 1b) show two different behaviors. Firstly, the extraction of Cu, Cd and Pb was positively correlated with pH, showing best efficacies at pH 8.0. The latter applies to all three TSILs and represents an important finding concerning the application in natural surface water samples, which usually lie between pH 6.5 and pH 8.5 [39]. At more acidic pH the protonation of the naphthoate group in the diffusion layer between the aqueous and the organic solution could hinder metal complexation, whereas with increasing pH this complexation could be enhanced because the deprotonated naphthoate group is free to form such complexes. This agrees with the results of Egorov et al., where extractable metal-salicylate complexes dissociated in acidic media due to the protonation of the functional salicylate anion [32]. The positive correlation of pH and extraction efficacy has also been reported for bis(2,4,4-trimethylpentyl)phosphinic acid extracting Zn, Co and Mn [40,41]. Secondly, Ag extraction remained constant at approx. 80% for all studied pH values, suggesting that an extraction mechanism differing from that of Cu, Cd and Pb predominated here. Future research should elucidate the exact extraction mechanisms and the respective roles of cations and the anion of these three TSILs.

Leaching was positively correlated with pH in all cases (Table S2). For [P_66614_][HNA], values increased from 2.9 ± 0.2 mg L^−1^ DOC at pH 2.0 to 11.3 ± 0.7 mg L^−1^ DOC at pH 8.0. A shift from neutral extraction to ion exchange at increasing pH values has been described for ILs by Janssen et al. [34]. This would explain the observed increased leaching at pH 8.0, under the premise that the cation of the applied TSIL is responsible for the measured DOC. Further studies are needed to confirm this. Based on the results, pH 8.0 was selected for subsequent experiments because it offered the highest extraction efficacies combined with an easy applicability for micro-extraction from natural alkaline samples. This makes sample modification, e.g., acidification, obsolete.

#### 2.1.3. Time Dependence

**Figure 2:**
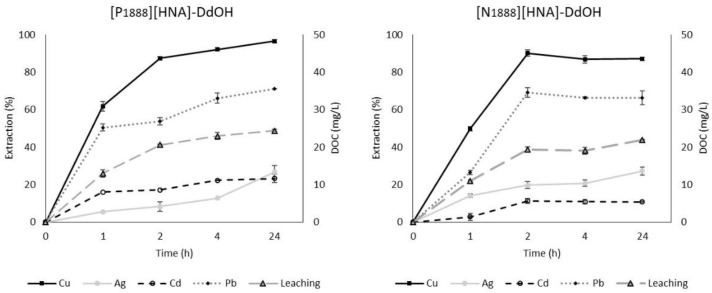
Time dependence of extraction and leaching using 50 wt % dodecan-1-ol (DdOH) mixtures of [P_1888_][HNA] and [N_1888_][HNA], respectively, pH = 8.0 (n = 3, error bars = ± SD).

Time dependence of extraction efficacy and leaching was evaluated for reaction times of 1, 2, 4 and 24 h (Figure 2 and Figure S1). For most metals, maximum extraction was achieved after 2 h using [P_1888_][HNA]-DdOH and [N_1888_][HNA]-DdOH. The setup of [N_1888_][HNA]-DdOH displayed the fastest extraction, reaching an extraction efficacy of 90.1 ± 1.7% for Cu after 2 h. This represents a 40% efficacy increase compared to using IL [A336][TS] in a single-metal SBME setup [29]. For the ILs used in this study, this improvement might be attributed to the higher polarity of [N_1888_][HNA] [30]. The overall highest extraction efficacies were achieved using [P_66614_][HNA]. For this TSIL, stabilization of extraction for all metals was reached after 4 h, halving the time required to achieve similar extraction compared with a liquid-liquid extraction setup for the same metals [30]. The superior surface/volume ratio of the fiber in SBME compared to a drop of the TSIL in liquid-liquid extraction could explain this effect. Furthermore, the time-dependent curve of extraction efficacy was similar for all investigated metals, suggesting a simultaneous and steady uptake. After 24 h, the SBs prepared with [P_66614_][HNA] simultaneously extracted 96.0 ± 0.3% of Ag, 93.5 ± 2.9% of Pb as well as 78.1 ± 2.7% of Cu and 45.8 ± 2.2% of Cd.

The influence of time on leaching increased continually during the first hour in all three cases. The lowest values were achieved by SBs prepared with [P_66614_][HNA]: between 7.8 ± 0.8 mg L^−1^ after 1 h and 15.8 ± 0.9 mg L^−1^ DOC after 24 h, displaying a stabilized curve after 4 h (13.8 ± 1.1 mg L^−1^). For the subsequent experiments, an extraction time of 2 h was chosen as it offered a good compromise between extraction efficacy and leaching as well as a projected technical viability.

#### 2.1.4. Stirring Rate

The influence of hydrodynamic conditions on extraction performance was investigated by modifying the stirring speed. The extraction efficacies are summarized in Table S1, leaching results in Table S2. Most notably, a 39.5 ± 0.7% extraction of Ag was already obtained in non-stirred samples for SBs prepared with [P_66614_][HNA]. In general, a positive correlation between stirring speed and extraction efficacy was observed. The effect was clearly evident when comparing non-stirred samples with samples stirred at 100 and 300 rpm, respectively, but was less pronounced at higher stirring rates. The exception was Pb, whose extraction efficacy still increased significantly between 300 and 600 rpm.

Leaching was only minimally affected by varying stirring rates. No stirring led to a measured DOC value of 7.9 ± 0.3 mg L^−1^ for [P_66614_][HNA], while values between 100 and 800 rpm remained almost identical, ranging between 10.9–11.7 mg L^−1^ DOC. Similar results were obtained for the other two TSILs, confirming the stability of SBME when using viscous TSILs. The stirring rate was set to 800 rpm for the subsequent experiments.

#### 2.1.5. Fiber Length

We studied the effect of varying volumes of the tested TSILs on extraction efficacy and leaching by changing fiber length. The extraction efficacy for 10 cm fibers was significantly reduced when compared to the initial length of 15 cm (Table S1). Increasing TSIL volume by using 20 cm fibers did not further enhance the efficacy. We therefore conclude that, under our experimental conditions, metal uptake following the concentration gradient between feed solution and TSIL was optimal at 15 cm fiber length. Moreover, elongating the SB to 20 cm had a negative effect on leaching. Specifically, leaching values were 7.0 ± 0.9 mg L^−1^ DOC for SBs prepared with 10 cm and 19.5 ± 1.8 mg L^−1^ DOC for 20 cm fibers in the case of [P_66614_][HNA]. Accordingly, the initial solvent bar size of 15 cm was retained for further experiments.

In summary, the selected conditions for the subsequent experimental setup were 15 cm fibers filled and impregnated with pure [P_66614_][HNA] or the 50 wt % dodecan-1-ol mixtures [P_1888_][HNA]-DdOH and [N_1888_][HNA]-DdOH, respectively, employed in samples at pH 8.0 which were stirred at 800 rpm for 2 h.

### 2.2. Synthetic Saline Samples

The obtained SBME setup was applied to feed solutions containing different NaCl concentrations to study the influence of rising salinity on extraction efficacy and leaching. The results were compared to previous works with other TSILs [42]. NaCl was added in concentrations from 5.0 g L^−1^ to 60.0 g L^−1^. The influence of counter ions on extraction performance has been well described in literature. CaCl_2,_ for example, has been used by Leyma et al. to increase the ionic strength, with chloride acting as a salting out agent, which increased the extraction efficacy and decreased leaching [14]. Messadi et al. reported a similar effect, where increasing concentrations of e.g., NaNO_3_ and NaCl led to reduced TOC values, which was attributed to a shift from cation exchange to ion pairing. In that case, the counter anion, e.g., nitrate, was co-extracted to maintain electroneutrality [38]. Lastly, different speciation of metals under saline conditions, namely the formation of negatively charged chlorido complexes [43], could impact the extraction performance as well.

**Figure 3:**
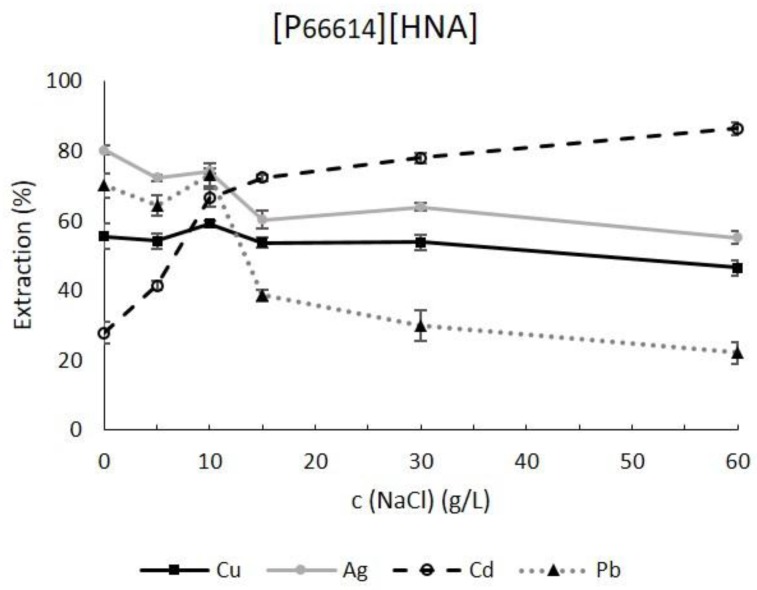
Effect of NaCl on extraction efficacy of solvent bars containing pure [P_66614_][HNA] at pH 8.0 for an extraction time of 2 h (n = 3, error bars = ± SD).

The most significant results were obtained for [P_66614_][HNA] (Figure 3). A strongly positive correlation between NaCl and extraction efficacy was recorded for Cd. This most probably reflects the formation of Cd chlorido complexes with increasing Cl^−^ concentration. CdCl_3_^−^ and CdCl_4_^2−^ have been reported as the prevalent species in Cd solutions containing 30–60 g L^−1^ NaCl [43]. Our results suggest that Cd chlorido complexes were extracted by anionic exchange of CdCl_n_^(n-2)−^ with naphthoate anions and/or a sacrificial ion exchange of Cl^−^, present as impurity in the TSIL [30] or coextracted from the feed solution as reported by Fischer et al. for similar TSILs [44]. Verifying these suggestions, however, will require further studying the roles of coexistent ions Na^+^ and Cl^−^ on the extraction performance in this particular case and subsequently determining the prevalent extraction mechanisms. Similar observations have been made for other ionic liquids such as methyltrioctyl-ammonium chloride [42]. Hence, we achieved a strong increase of extraction efficacy from 27.9 ± 3.2% for the feed solution without NaCl to 86.5 ± 1.9% for the hypersaline feed solution with 60 g L^−1^ NaCl.

Nonetheless, a positive correlation between extraction efficacy and ionic strength by adding NaCl could not be found for all metals. Opposite effects were recorded for Pb extraction, which significantly decreased with more than 10 g L^−1^ NaCl in the sample. While the efficacy up to 10 g L^−1^ was approx. 70%, a strong decrease to 38.6 ± 1.6% occurred for 15 g L^−1^, further decreasing to 22.1 ± 3.1% for 60 g L^−1^ NaCl (Figure 3). PbCl_n_^(n−2)−^ complexes are prevalent under marine saline conditions [45]. The differences in extraction behavior compared to Cd warrant further investigations. Cu extraction was almost unaffected, with efficacies between 47 and 59%, possibly indicating an extraction of the free metal ion based on complexation rather than ion exchange. Examining the differences in efficacies under saline conditions for Ag and Cd, the enhanced values for Cd could stem from the formation of differently charged chlorido complexes. During anion exchange, more monovalent CdCl_3_^−^ molecules can be exchanged compared to the bivalent AgCl_3_^2−^ [42].

In the experiments with [P_1888_][HNA]-DdOH and [N_1888_][HNA]-DdOH, efficacy declined for all metals (Table S1).

Concerning leaching, rising NaCl content led to insignificant effects for the three TSILs (Table S2). For [P_66614_][HNA], values ranged from 10.2 mg L^−1^ to 16.1 mg L^−1^ DOC, for [P_1888_][HNA]-DdOH from 16.7 mg L^−1^ to 24.4 mg L^−1^ DOC and for [N_1888_][HNA]-DdOH from 12.3 mg L^−1^ to 19.4 mg L^−1^. This repeatedly highlights consistent stability under different salinities and points to neutral extraction as the prevalent mechanism for the highly hydrophobic TSILs used in this work [35,38].

These results demonstrate that our setup with a SB impregnated with [P_66614_][HNA] constituted a promising alternative for the micro-extraction of Cd from saline and hypersaline samples, for which only a limited number of reagents have proved to be efficient.

### 2.3. Applicability in Natural Water Samples

After investigating the capability to extract the selected heavy metals as well as the leaching behavior under different experimental parameters and feed solution salinities, we conducted experiments with spiked natural water feed solutions.

**Figure 4:**
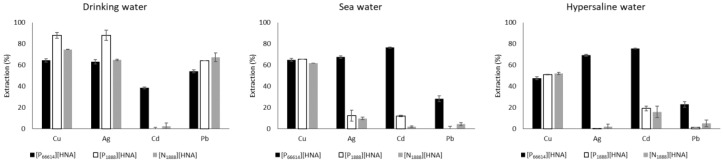
Extraction efficacies of the SBME setups using pure [P_66614_][HNA] and the two respective 50 wt % dodecan-1-ol mixtures of [P_1888_][HNA] and [N_1888_][HNA] in natural water feed solutions for an extraction time of 2 h (n = 3, error bars = ± SD).

In general, extraction and leaching results (Figure 4 and Table S2) agreed well with data obtained from synthetic feed solutions. For drinking water, SBs containing [P_1888_][HNA]-DdOH simultaneously extracted 88.0 ± 2.8% of Cu, 87.9 ± 4.8% of Ag and 64.3 ± 0.2% of Pb within 2 h. This displays a promising capability for employing this setup in e.g., the preconcentration of trace metals from freshwater matrices. Additionally, leaching remained within the values obtained for pure water feed solutions. Thus, we were able to improve the extraction performance in spiked drinking water compared to first batch experiments using different TSILs in SBME [29]. Importantly, this is based on a multi-elemental approach as opposed to single metal experiments, lower leaching and, in the case of Cu, Ag and Pb, shorter extraction times.

Regarding sea water, Cu was extracted similarly well by all three TSILs (approx. 65% efficacy). As observed for the Cl^−^ dependent experiments, [P_66614_][HNA] demonstrated an enhanced extraction performance for Ag and Cd, with efficacies of 67.5 ± 1.1% and 76.5 ± 0.5%, respectively. Pb extraction, in contrast, was equally inhibited under natural saline conditions as it was for the synthetic feed solution containing 30 g L^−1^ NaCl.

For the hypersaline feed solution, a similar decrease in Cu extraction occurred for all setups compared to natural sea water. This stands in contrast to the results for synthetic feed solutions, where significantly decreasing efficacies in the range of 30.0–60.0 g L^−1^ NaCl were mainly observed for [P_1888_][HNA]-DdOH. We explain this decrease down to an overall efficacy of approx. 50% by the higher DOC value of the hypersaline water (9.1 mg L^−1^) compared to sea water (2.2 mg L^−1^): extraction efficacies of Cu are highly affected by the presence of organic matter. Cu displays a high affinity towards dissolved organic matter in general and humic acids in particular. The resulting strong complexes hinder extraction [46,47]. The efficient extraction of Ag and Cd for [P_66614_][HNA] recorded for sea water samples remained intact, with values of 69.2 ± 0.8% and 75.6 ± 0.2%, respectively. In both cases, extractable chlorido complexes rather than DOC complexes are formed. This makes Cd and Ag extraction insensitive to the presence of organic matter in saline samples. These results are in good accordance with results reported by Herce-Sesa et al. [42] for a different SBME setup.

As observed for the drinking water feed solution, the stability of the SBME setups also prevailed under natural saline and hypersaline conditions. Moreover, the leaching values were the same as those for pure water (Table S2).

Lastly, the extraction efficacies for the wastewater treatment plant effluent feed solution were considerably decreased for all metals. Moderate extraction was recorded for Cu, with 30–39% extracted for the three TSILs, and a 35.5 ± 2.3% extraction of Cd using [P_66614_][HNA], whereas Ag and Pb extraction was inhibited in all cases. As the composition of the effluent was not determined in detail, we speculate that undefined contents, e.g., chelating agents or natural organic matter with high sulfur and nitrogen contents [48], formed strong, unextractable complexes with the spiked metals.

## 3. Materials and Methods

### 3.1. Solvents and Reagents

Standard solutions of the metals Cu, Ag, Cd and Pb—1000 mg L^−1^ in 2–4% (*w*/*w*) HNO_3_ (Sigma-Aldrich, St. Louis, MO, USA)—were used to prepare feed solutions and calibrate atomic absorption spectroscopy. Ionic liquids [P_66614_][HNA], [P_1888_][HNA] and [N_1888_][HNA] were synthesized and characterized as described elsewhere [30]. For the preparation of feed solutions, sodium chloride (99%), nitric acid (p.a., 65%) and sodium hydroxide (98%) from Panreac (Barcelona, Spain) were used. The organic solvents tested for dissolving the TSILs were kerosene (purum, Sigma-Aldrich), octan-1-ol (ACS-reagent, ≥99%, Sigma-Aldrich) and dodecan-1-ol (97%, Buchs, Switzerland). Ultra-pure water of resistivity < 18.2 MΩ cm was obtained from a Millipore Milli-Q Academic apparatus (Merck Millipore, Burlington, MA, USA).

### 3.2. Instrumentation

Metals in the samples were quantified by high-resolution continuum source flame atomic absorption spectrometer contrAA^®^ 700 (Analytik Jena AG, Jena, Germany). This instrument was set up for a sequential, multi-elemental measurement of all applied metals, measuring the following wavelengths: Cu 324.75 nm, Ag 328.07 nm, Cd 228.80 nm and Pb 217.00 nm. An acetylene/air flame was utilized to atomize the samples, and the spectrometer detector was set up between pixels 98–102. Dissolved organic carbon (DOC) was measured using a multi N/C 3100 analyzer (Analytik Jena AG).

To determine pH values, a BasiC 20 pH meter was utilized, and conductivity of the samples was measured with a BasiC30 conductometer (Crison, Barcelona, Spain). Mass concentrations of NaCl were calculated from the chloride content measured with the ion selective electrode pH & ION meter GLP 22^+^ (Crison). Samples were stirred with Labbox instruments H01 series magnetic stirrers (Labbox, Barcelona, Spain).

### 3.3. Preparation of Solvent Bars (SB) and Setup of Extraction Experiments

In this study, polypropylene Accurel PP S6/2 hollow fibers (pore size 0.2 μm, internal diameter 1800 μm; Membrana, Wuppertal, Germany) were used as support for the TSILs. Hollow fibers were prepared in the form of a solvent bar as previously reported in the literature [42]. For this purpose, 15 cm of fiber were cut and thermally sealed on one end. Organic additives were used to liquefy the two TSILs that were solid at room temperature. Using a syringe, the fiber lumen and the pores were filled with the TSIL or TSIL mixed with organic solvent. Thereafter, the other fiber end was thermally sealed and the SB was rinsed with water to eliminate possible excess TSIL in the fiber wall. Finally, the SB was deployed into 50 mL of feed solution during the respective extraction time. All experiments were conducted in triplicates at 25 ± 0.1 °C, stabilized in a climate box (VELP Scientifica, Usmate Velate, Italy). To consider metal loss due to possible oxidation-, precipitation- and adhesion effects, reference samples were prepared additionally. These samples consisted of only 50 mL feed solution and were treated equally to samples containing SBs.

### 3.4. Feed Solutions and Sample Conditions

#### 3.4.1. Influence of Physico-Chemical Properties on SBME

The synthetic feed solution used in these experiments consisted of pure water (Millipore) spiked with 1 mg L^−1^ Cu, Ag and Cd as well as 4 mg L^−1^ Pb. An initial pH of 8.0 was chosen because the utilized TSILs displayed good extraction capabilities in liquid-liquid and solid-liquid extraction at this pH [30]. The experimental parameters for each step are described in the following paragraphs.

##### Organic Additives

In a preliminary test, we investigated the effects of different organic additives utilized in previous works [42,49] on extraction efficacy and leaching. The three studied additives were octan-1-ol, dodecan-1-ol and a 4:1 mixture of kerosene and dodecan-1-ol. Samples were stirred at 600 rpm for 2 h (the initial conditions for all experiments). Out of the tested solutions, dodecan-1-ol was chosen for the subsequent experiments because it offered the lowest leaching combined with equally high extraction efficacies.

The effect of adding dodecan-1-ol on the extraction performance was then studied by adding 75, 50 and 25 wt % dodecan-1-ol to [P_66614_][HNA] and comparing it to the pure TSIL. For [P_1888_][HNA] and [N_1888_][HNA], a feasible maximum percentage of 50 wt % dodecan-1-ol was determined, limiting the used concentration range to 90, 75, 60 and 50 wt % dodecan-1-ol, because at lower concentrations the SBs became too brittle to handle. Again, samples at pH 8.0 were stirred at 600 rpm for 2 h.

##### Feed Solution pH

The feed solution pH was adjusted to values of 2.0, 4.0, 6.0 and 8.0, respectively, by adding nitric acid or sodium hydroxide solution. SBs with pure TSIL [P_66614_][HNA] or [P_1888_][HNA] and [N_1888_][HNA] with 50 wt % dodecan-1-ol, respectively, were stirred for 2 h with 600 rpm.

##### Time Dependence

In order to study the time-dependent progression of the extraction, SBs using pure TSIL [P_66614_][HNA] and [P_1888_][HNA] as well as [N_1888_][HNA] with 50 wt % dodecan-1-ol, respectively, were employed in feed solution at pH 8.0 with a stirring rate of 600 rpm and durations of 1, 2, 4 and 24 h.

##### Stirring Rate

The effect of stirring rate on extraction efficacy and leaching was examined for unstirred samples and stirring rates of 100, 300, 600 and 800 rpm using pure [P_66614_][HNA] or [P_1888_][HNA] and [N_1888_][HNA] with 50 wt % dodecan-1-ol, respectively, with a feed solution pH of 8.0 for an extraction time of 2 h.

##### Fiber Length

We varied fiber length to evaluate a possible influence of the TSIL volume. 10 cm and 20 cm fibers were used to prepare SBs (versus initial length of 15 cm). Pure TSIL [P_66614_][HNA] or TSILs [P_1888_][HNA] and [N_1888_][HNA], with 50 wt % dodecan-1-ol, respectively, were used to prepare SBs. The feed solution was adjusted to pH 8.0 and samples were stirred at 800 rpm for 2 h.

#### 3.4.2. Synthetic Saline Samples

Saline synthetic feed solutions were prepared by spiking the pure water feed solution with 5, 10, 15, 30 and 60 g L^−1^ NaCl, respectively, and adjusting the pH to 8.0. The setup used 15 cm SBs containing the pure TSIL [P_66614_][HNA] or [P_1888_][HNA] and [N_1888_][HNA] with 50 wt % dodecan-1-ol, respectively, stirring the samples at 800 rpm for 2 h.

#### 3.4.3. Natural Water Samples

Finally, in order to evaluate the effect of natural sample matrices on the method’s performance, the selected setups were applied to the simultaneous micro-extraction of Cu, Ag, Cd and Pb from spiked natural water feed solutions. These setups consisted of 15 cm SBs prepared with pure [P_66614_][HNA] or [P_1888_][HNA] and [N_1888_][HNA] with 50 wt % dodecan-1-ol, respectively, stirred at 800 rpm for 2 h.

The natural water samples were taken from the following sites in Cádiz, southwest Spain: drinking water from the University of Cádiz Campus Puerto Real (“drinking water”), the effluent of wastewater treatment plant EDAR El Trocadero (“WWTP effluent”), sea water from Río San Pedro (“sea water”, collected at 36.531581, −6.215401), as well as a hypersaline sample (“hypersaline water”, collected at 36.533541, −6.210937). Prior to spiking, the natural samples were filtered using 0.45 µm nylon microfiltration membranes (Dorsan, Spain). The respective parameters of the natural water samples are summarized in Table 1. Measured parameters of drinking water were well comparable to data provided by the Spanish authorities [50].

### 3.5. Quantification

The extraction efficacy was calculated as the percentage of metal removed from the feed solution after the extraction experiment compared to the concentration measured in reference samples (Equation 1), where *C_Ref_* is the concentration of metal in the reference samples and *C_t_* is the metal concentration measured after extraction.

(1) $$\;Extraction\;efficacy\;\left(\%\right)=\frac{C_{Ref}-C_{t}}{C_{Ref}}\times 100\;$$

The leaching of TSIL or the TSIL mixed with organic solvent into the sample was measured as dissolved organic carbon (DOC) released during extraction.

## 4. Conclusions

The recently synthesized TSILs trihexyltetradecylphosphonium-([P_66614_]-), methyltrioctyl-phosphonium-([P_1888_]-) and methyltrioctylammonium 3-hydroxy-2-naphthoate ([N_1888_][HNA]) were successfully tested in a two-phase configuration of solvent-bar micro-extraction using porous, hollow polypropylene fibers as support. The metals Cu, Ag, Cd and Pb were simultaneously extracted under varying conditions.

Extraction efficacy was positively correlated with pH, with highest efficacies at pH 8.0. We therefore successfully demonstrated the SBME of heavy metals from natural waters without the need for sample pretreatment or modification. Moreover, TSIL stability was superior to similar SBME setups utilizing other TSILs. We attribute the low leaching values in all feed solution compositions to the increased hydrophobicity of the TSILs used.

SBs containing [P_66614_][HNA] showed best results for the simultaneous extraction of Ag and Cd from saline and hypersaline (≤60 g L^−1^ NaCl) water samples, possibly due to an efficient extraction mechanism of prevalent chlorido species. Using SBs impregnated with [P_1888_][HNA] in 50% dodecan-1-ol already yielded high efficacies for Cu and Ag (both 88%) as well as Pb (64%) from a drinking water feed solution after 2 h.

The novel combination of 3-hydroxy-2-naphthoate TSILs with SBME setups displayed high extraction efficacies, in many cases unaffected by sample composition and metal speciation, as well as high stability. This represents a significant improvement over earlier studies. These results call for further developing this simple and ecofriendly method for extracting trace metals from natural waters without sample modification. Future work could also implement this system in trace metal analysis, e.g., as a means of preconcentrating and subsequently directly measuring the metal-containing solvent bars.

## Figures and Tables

**Table 1:** Composition of collected natural water samples used for extraction experiments. WWTP: wastewater treatment plant, DOC: dissolved organic carbon.

Sample	pH	NaCl (g L^−1^)	Conductivity (mS cm^−1^)	DOC (mg L^−1^)
Drinking water	7.88	0.04	0.56	0.6
WWTP effluent	7.86	0.59	1.82	12.2
Sea water	8.06	36.4	42.9	2.2
Hypersaline water	8.17	55.9	55.9	9.1

## Supplementary Materials

The following are available online, Table S1: Summarized results for the optimization of extraction efficacy. Considered were all metals that showed an extraction efficacy >40% after the time dependent experiments for each setup respectively; Table S2: Summarized results for leaching during extraction; Figure S1: Time dependency of extraction and leaching using pure [P_66614_][HNA], pH = 8.0 (n = 3, error bars = ± SD). Summarized results for extraction efficacy and leaching are given in Tables S1 and S2, the time-dependent extraction and leaching behavior of [P_66614_][HNA] in Figure S1.
