# Supplementary material for: Solvent Bar Micro-Extraction of Heavy Metals from Natural Water Samples Using 3-Hydroxy-2-Naphthoate-Based Ionic Liquids
Source: Molecules. 2018 Nov 17;23(11):3011. doi: 10.3390/molecules23113011 (PMC6278406; doi:10.3390/molecules23113011)
Supplement: Supplementary file 1 [file molecules-23-03011-s001.pdf]

Supplementary information for:

Solvent bar micro-extraction of heavy metals from natural water samples using 3-hydroxy-2-naphthoate-based ionic liquids

Philip Pirkwieser[1,2], José A. López-López[2], Wolfgang Kandioller[1], Bernhard K. Keppler[1], Carlos Moreno[2] and Franz Jirsa[1,3]*

[1] Institute of Inorganic Chemistry, Faculty of Chemistry, University of Vienna, Waehringer Strasse 42, 1090 Vienna, Austria
[2] Department of Analytical Chemistry, Faculty of Marine and Environmental Sciences, Instituto de Investigación Marina (INMAR), University of Cádiz, PC 11510, Puerto Real, Spain
[3] Department of Zoology, University of Johannesburg, PO Box 524, Auckland Park, 2006, Johannesburg, South Africa
* Correspondence: franz.jirsa@univie.ac.at

Table of contents

Table S1: Summarized results for the optimization of extraction efficacy. Considered were all metals that showed an extraction efficacy >40% after the time dependent experiments for each setup respectively.

Table S2: Summarized results for leaching during extraction.

Figure S1: Time dependency of extraction and leaching using pure [P66614][HNA], pH = 8.0 (n=3, error bars = ±SD).

Table S1: Summarized results for the optimization of extraction efficacy. Considered were all metals that showed an extraction efficacy >40% after the time dependent experiments for each setup respectively.

		Extraction efficacy ± SD (%)							
		**Cu**			**Ag**	**Cd**	**Pb**		
		**[P$_{66614}$][HNA]**	**[P$_{1888}$][HNA]**	**[N$_{1888}$][HNA]**	**[P$_{66614}$][HNA]**	**[P$_{66614}$][HNA]**	**[P$_{66614}$][HNA]**	**[P$_{1888}$][HNA]**	**[N$_{1888}$][HNA]**
**Dodecan-1-ol (wt%)**	90	n/a	45.6 ± 1.3	29.5 ± 1.6	n/a	n/a	n/a	28.1 ± 1.7	12.9 ± 0.8
	75	<5	70.4 ± 4.7	40.0 ± 7.0	35.2 ± 2.9	5.5 ± 0.9	<5	40.8 ± 2.8	37.7 ± 8.3
	60	n/a	83.7 ± 2.5	80.3 ± 1.4	n/a	n/a	n/a	47.6 ± 0.8	47.3 ± 6.8
	50	<5	87.4 ± 0.8	90.1 ± 1.7	73.8 ± 2.5	4.4 ± 0.4	20.9 ± 6.7	50.4 ± 2.1	69.1 ± 2.7
	25	30.6 ± 2.8	n/a	n/a	74.8 ± 2.6	9.1 ± 3.0	54.3 ± 3.0	n/a	n/a
	0	55.5 ± 3.7	n/a	n/a	80.2 ± 1.2	27.9 ± 3.2	70.1 ± 3.4	n/a	n/a
**pH**	2	<5	<5	<5	76.9 ± 0.9	<5	<5	<5	<5
	4	33.8 ± 2.1	<5	<5	83.2 ± 1.0	11.4 ± 1.6	55.8 ± 2.8	16.8 ± 1.9	<5
	6	54.0 ± 3.4	37.6 ± 3.6	56.1 ± 1.0	82.4 ± 1.3	18.9 ± 1.2	64.7 ± 3.5	15.5 ± 2.4	32.5 ± 3.3
	8	55.5 ± 3.7	87.4 ± 0.8	90.1 ± 1.7	80.2 ± 1.2	27.9 ± 3.2	70.1 ± 3.4	50.4 ± 2.1	69.1 ± 2.7
**Stirring rate (rpm)**	0	17.3 ± 0.6	15.5 ± 4.7	28.2 ± 2.9	39.5 ± 0.7	15.4 ± 0.3	11.6 ± 1.4	19.1 ± 2.2	16.1 ± 1.2
	100	42.4 ± 1.1	59.4 ± 0.8	61.0 ± 0.9	53.3 ± 1.8	23.2 ± 0.4	48.6 ± 1.6	32.6 ± 1.3	35.0 ± 1.7
	300	56.8 ± 1.0	75.1 ± 0.1	85.6 ± 1.8	73.5 ± 0.4	29.4 ± 0.4	56.4 ± 2.0	44.9 ± 2.0	73.3 ± 3.1
	600	55.5 ± 3.7	87.4 ± 0.8	90.1 ± 1.7	80.2 ± 1.2	27.9 ± 3.2	70.1 ± 3.4	50.4 ± 2.1	69.1 ± 2.7
	800	63.1 ± 0.0	74.1 ± 4.3	87.8 ± 3.5	81.4 ± 1.4	27.2 ± 0.2	70.7 ± 3.6	57.5 ± 3.0	68.0 ± 4.4
**Fiber length (cm)**	10	45.7 ± 0.2	72.3 ± 3.8	75.7 ± 0.8	70.9 ± 1.1	14.5 ± 2.5	62.2 ± 1.6	29.9 ± 1.7	54.7 ± 2.1
	15	55.5 ± 3.7	87.4 ± 0.8	90.1 ± 1.7	80.2 ± 1.2	27.9 ± 3.2	70.1 ± 3.4	50.4 ± 2.1	69.1 ± 2.7
	20	57.4 ± 0.9	88.7 ± 1.4	82.7 ± 1.5	81.8 ± 0.6	27.1 ± 2.8	65.3 ± 2.0	39.5 ± 3.2	61.3 ± 0.9
**NaCl (g L^{-1})**	0	55.5 ± 3.7	87.4 ± 0.8	90.1 ± 1.7	80.2 ± 1.2	27.9 ± 3.2	70.1 ± 3.4	50.4 ± 2.1	69.1 ± 2.7
	5	54.2 ± 2.3	77.9 ± 3.7	50.1 ± 1.9	72.3 ± 0.8	41.4 ± 1.2	64.5 ± 2.9	31.6 ± 1.9	19.3 ± 2.3
	10	59.1 ± 1.1	79.7 ± 3.7	53.3 ± 1.5	74.2 ± 0.7	66.5 ± 2.6	73.1 ± 3.2	39.7 ± 2.6	18.4 ± 1.4
	15	53.7 ± 1.5	60.7 ± 3.7	56.3 ± 2.0	60.3 ± 2.6	72.3 ± 0.9	38.6 ± 1.6	21.6 ± 2.8	26.4 ± 0.6
	30	53.8 ± 2.3	77.2 ± 1.5	47.2 ± 1.4	63.9 ± 1.2	78.0 ± 1.6	29.9 ± 4.2	<5	8.8 ± 0.6
	60	46.6 ± 2.2	36.4 ± 2.0	40.6 ± 2.0	55.2 ± 1.8	86.5 ± 1.9	22.1 ± 3.1	<5	9.9 ± 1.5

n/a= not applicable.

Table S2: Summarized results for leaching during extraction. WWTP = wastewater treatment plant, DOC = dissolved organic carbon.

		Leaching ± SD (mg L^{-1} DOC)		
		**[P_{66614}][HNA]**	**[P_{1888}][HNA]**	**[N_{1888}][HNA]**
**Dodecan-1-ol (wt%)**	90	n/a	14.9 ± 1.4	6.2 ± 0.9
	75	11.6 ± 0.6	19.3 ± 0.9	9.0 ± 0.2
	60	n/a	21.1 ± 1.1	15.6 ± 0.8
	50	13.9 ± 0.1	20.6 ± 0.2	19.4 ± 0.6
	25	12.8 ± 0.1	n/a	n/a
	0	11.3 ± 0.7	n/a	n/a
**pH**	2	2.9 ± 0.2	12.6 ± 0.3	8.0 ± 0.3
	4	7.6 ± 0.5	16.6 ± 0.9	13.2 ± 0.2
	6	10.1 ± 0.5	18.2 ± 0.6	15.1 ± 0.9
	8	11.3 ± 0.7	20.6 ± 0.2	19.4 ± 0.6
**Stirring rate (rpm)**	0	7.9 ± 0.3	16.2 ± 0.9	13.3 ± 0.5
	100	10.9 ± 0.6	22.8 ± 1.2	18.3 ± 1.0
	300	11.4 ± 0.4	21.7 ± 1.8	18.7 ± 0.3
	600	11.3 ± 0.7	20.6 ± 0.2	19.4 ± 0.6
	800	11.7 ± 0.4	24.8 ± 2.2	19.9 ± 0.6
**Fiber length (cm)**	10	7.0 ± 0.9	17.5 ± 1.2	14.9 ± 0.2
	15	11.3 ± 0.7	20.6 ± 0.2	19.4 ± 0.6
	20	19.5 ± 1.8	24.5 ± 0.9	21.0 ± 0.5
**NaCl (g L^{-1})**	0	11.3 ± 0.7	20.6 ± 0.2	19.4 ± 0.6
	5	13.9 ± 0.3	19.2 ± 0.9	12.3 ± 0.5
	10	16.1 ± 0.5	21.6 ± 0.9	14.5 ± 0.1
	15	13.9 ± 0.2	16.7 ± 0.7	14.7 ± 0.1
	30	10.2 ± 0.1	23.4 ± 1.9	11.2 ± 0.2
	60	12.6 ± 0.4	24.4 ± 2.1	14.7 ± 0.2
**Drinking water**		16.2 ± 1.3	24.5 ± 2.0	19.4 ± 0.5
**WWTP effluent**		3.8 ± 0.3	25.4 ± 0.4	19.3 ± 0.2
**Sea water**		12.2 ± 0.3	29.7 ± 0.1	26.1 ± 2.7
**Hypersaline water**		11.3 ± 0.9	27.0 ± 0.0	24.6 ± 0.5

n/a= not applicable.

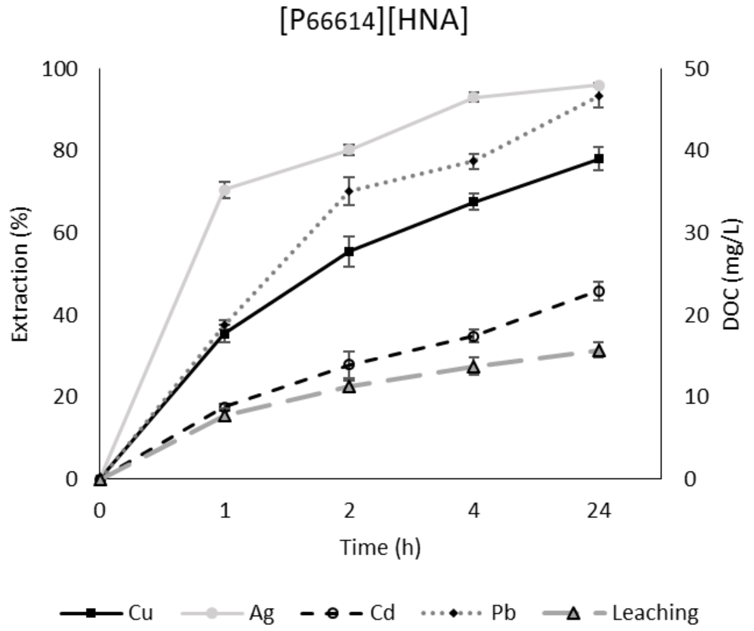

Figure S1: Time dependency of extraction and leaching using pure [P_{66614}][HNA], pH = 8.0 (n=3, error bars = ± SD).
